# ACE: A Consent-Embedded privacy-preserving search on genomic database

**DOI:** 10.1016/j.heliyon.2024.e29399

**Published:** 2024-04-16

**Authors:** Sara Jafarbeiki, Amin Sakzad, Ron Steinfeld, Shabnam Kasra Kermanshahi, Chandra Thapa, Yuki Kume

**Affiliations:** a Monash University, Australia; b CSIRO, Australia; c University of New South Wales (UNSW) Canberra, Australia

**Keywords:** Dynamic searchable encryption, Genomic data privacy, Secure outsourcing, Querying of encrypted data, Cloud security

## Abstract

In this paper, we introduce ACE, a consent-embedded searchable encryption scheme. ACE enables dynamic consent management by supporting the physical deletion of associated data at the time of consent revocation. This ensures instant real deletion of data, aligning with privacy regulations and preserving individuals' rights. We evaluate ACE in the context of genomic databases, demonstrating its ability to perform the addition and deletion of genomic records and related information based on ID, which especially complies with the requirements of deleting information of a particular data owner. To formally prove that ACE is secure under non-adaptive attacks, we present two new definitions of forward and backward privacy. We also define a new hard problem, which we call D-ACE, that facilitates the proof of our theorem (we formally prove its hardness by a security reduction from DDH to D-ACE). We finally present implementation results to evaluate ACE's performance.

## 1 Introduction

The rapid advancements in the generation and accessibility of genomic data have significantly influenced scientific studies, providing unprecedented opportunities to delve into the intricate connections between diseases and genes. The wealth of genomic datasets has enabled researchers to uncover valuable insights into the genetic basis of various conditions. However, with the vastness of these datasets, traditional computational resources often fall short in efficiently processing and analysing the wealth of information present.

To address the challenges posed by the enormous genomic data, cloud servers have emerged as a powerful solution. By leveraging cloud computing capabilities, researchers can tap into the extensive computing power and storage capacity of cloud infrastructures. In genomics research, prioritizing the ethical treatment of study participants is crucial. As the genomic data involved can carry sensitive and deeply personal information, researchers must prioritize data privacy and participant consent. The concept of dynamic informed consent has gained prominence, emphasising the importance of ongoing communication between researchers and study participants. By keeping participants informed about the study's objectives, potential risks, and their right to withdraw their consent at any stage, dynamic informed consent fosters a more transparent and respectful research environment [1], [2].

However, genomic data privacy goes beyond mere participant consent. Genomic information is unique and, once revealed, irreversible, carrying potential stigmatizing effects for both individuals and their families. Therefore, safeguarding genomic data from unauthorised access and ensuring its privacy become paramount concerns. The potential misuse of genomic data extends beyond legal consequences, posing significant threats to personal privacy and ethical principles. Unauthorized access to this sensitive information could result in discrimination, stigmatization, and various ethical breaches. Genetic data, offering insights into health and ancestry, becomes vulnerable to misuse, potentially leading to identity theft and fraudulent activities. These ethical and societal concerns not only affect individuals but also jeopardize the credibility of genetic research, undermining public trust and deterring participation in vital studies. Cloud platforms, while offering numerous benefits, also present potential security risks if not appropriately managed. Storing sensitive genetic data on public cloud servers demands robust privacy and security measures to avoid any unintended disclosures or breaches [3], [4].

Moreover, encrypted search schemes should comply with the requirements outlined in the General Data Protection Regulation (GDPR) [5], which grants individuals the entitlement to request the erasure and cessation of processing of their personal data when such data are no longer essential for the objectives for which they were initially gathered or processed, and the organisation must stop the processing of individuals' data and delete them as soon as an individual withdraws their consent [5]. This requires instant deletion of data. To protect genomic data stored in the cloud and maintain its searchability, searchable encryption has emerged as a promising cryptographic technique. In particular, dynamic searchable symmetric encryption (DSSE) enables alterations to the encrypted database while ensuring the ability to conduct searches. However, existing DSSE schemes face challenges in achieving instant non-interactive real deletion of data when participants revoke their consent. Furthermore, certain schemes inadvertently reveal identifiers (IDs) during the update phase, compromising privacy [6], [7], [8].

Table 1 provides an overview of DSSE schemes, focusing on their behaviour in deleting ID information, privacy considerations, and communication cost. Deletion can be (i) based on an identifier (ID) or a keyword (w); (ii) it can be physical or logical,^1^ (iii) with instant deletion meaning removing data upon request. Some schemes keep data on the server and delete it at a later time (e.g., when a search on data happens), making them unsuitable for immediate consent revocation (non-instant). (iv) Non-interactive deletion is achieved with one deletion token based on ID. Privacy considerations include (v) forward and backward privacy, as well as (vi) ID privacy. (vii) Communication cost refers to the size of the token for deletion. However, no existing scheme fulfils all these requirements, making it a challenging area for further research. Table 1 provides a detailed overview of DSSE schemes and highlights the existing limitations and challenges in current encryption schemes, underscoring the need for innovative approaches, such as the one proposed in this paper, to address these shortcomings and contribute to the advancement of genomic data security. Moreover, in regards to theoretical underpinnings, insights from Table 1 indicate that previous works do not fully meet the specific requirements and goals for our application (as shown in the first row of Table 1: non-interactive instant physical deletion based on ID while preserving privacy).

**Table 1:** Existing dynamic searchable symmetric encryption schemes comparison.

Scheme	Deletion				Privacy		Comm. cost^§^
	Approach based on	Type	Instant	Non-interactive^‡^	FP/BP	ID	
[9]	w	Logical	✗	✗	FP/BP	✗	O(x)
[10]	w	Logical	✗	✗	FP/BP	✗	O(x)
[11]	w	Logical	✗	✗	FP	✗	O(xlog⁡(rx))
[12]	ID	Physical	✗	✓	-^⁎⁎^	✗	O(1)
[13]	w	Physical	✗	✗	FP/BP	✓^⁎^	O(x)
[14]	w	Logical	✗	✗	FP/BP	✗	O(x)
ACE	ID	Physical	✓	✓	IDFP/IDBP^⁎⁎⁎^	✓	O(1)

Notations: FP: Forward Privacy; BP: Backward Privacy; *x*: Number of keywords of an ID; *r*: Number of records (IDs) in DB; ^§^: Communication cost is compared for the deletion phase when the information of an ID needs to be deleted; ^‡^: When the data of an ID is deleted; ^⁎⁎^: FP/BP have not been discussed in this paper; ^⁎⁎⁎^: Please review section 5 for the definitions and further details; ^⁎^: Their leakage model does not formalize this privacy.

In this research, our goal is to provide the above-identified requirements based on (i) to (vii) factors (Non-interactive instant physical deletion based on ID with privacy considerations and low communication cost) altogether while focusing on the challenge of securely outsourcing genomic data to cloud servers and maintaining data privacy. To achieve this, we propose a novel approach that treats each piece of genomic information, such as Single Nucleotide Polymorphisms (SNPs) and phenotype data, as a separate keyword linked with a unique identifier (ID). By doing so, we can conduct searches on individual genomic pieces as distinct keywords, thereby preserving data privacy without directly exposing the actual data. The contributions outlined in this paper are:

– As the first theoretical contribution, we propose a novel construction named ACE that facilitates efficient keyword-based search and non-interactive addition/deletion operations based on identifiers (IDs). By treating each genomic piece as a separate keyword associated with a unique ID, ACE allows searches without directly revealing the underlying data. The deletion process in ACE operates based on ID, requiring only a single token to remove all corresponding entries for that ID physically. This approach minimizes communication costs during delete operations and ensures a non-interactive and prompt deletion process. Unlike other schemes that may retain data until a search occurs, ACE achieves instant deletion without undue delay. This capability aligns with the GDPR requirements.
– As the second theoretical contribution, our structure enables search based on keywords and deletion based on IDs, leading to the introduction of two new privacy notions: IDFP (ID-based Forward Privacy) and IDBP (ID-based Backward Privacy). These notions capture privacy in dynamic SSE with updates based on IDs, distinct from conventional mechanisms with search and update based on keyword and ID pairs. We rigorously prove that ACE achieves privacy under non-adaptive attacks in accordance with the IDFP and IDBP notions, relying on the hardness of the Decisional Diffie-Hellman (DDH) problem. To facilitate the proof, we introduce an intermediate computational problem called Decisional-ACE (D-ACE) to support our analysis, showing that the hardness of D-ACE follows from the hardness of DDH.
– In the third contribution, we present comprehensive implementation results of ACE on genomic datasets, evaluating its performance in terms of the update and search computation costs, communication costs, and storage. The results demonstrate that ACE exhibits high performance and efficiency while delivering all the previously mentioned features. Although primarily designed for genomic data applications, ACE's versatility allows its application as an ID-based DSSE in other scenarios where update operations based on IDs are necessary.

### 1.1 Related works

Song et al. [26] pioneered the use of symmetric key encryption to address the challenge of conducting keyword searches over encrypted data, a concept referred to as searchable symmetric encryption (SSE). Nevertheless, its search time is directly proportional to the number of keyword and identifier pairs, posing limitations on efficiency. Subsequently, Goh [27] proposed a secure indexing technique aimed at improving search efficiency by achieving linear search time with respect to the number of files. Building upon this, Curtmola et al. [28] introduced SSE with sublinear search time leveraging an inverted index data structure. Additionally, they formalized the security model of SSE (as Real vs. Ideal), a framework widely embraced in subsequent research. Over time, numerous SSE schemes with diverse enhancements have emerged [23], [29], [30], [31]. SSE has also been studied to provide privacy-preserving query execution over genomic databases [32], [33], [34]. However, these schemes are not dynamic.

To address the need for updates in searchable symmetric encryption (SSE), researchers have proposed dynamic SSE (DSSE) schemes [35], [36]. However, these methods may inadvertently disclose additional information during updates, potentially compromising data privacy. An alternative approach, exemplified by schemes such as [37], involves the server operating solely as an entity for transmission and storage, thereby minimizing information leakage. Nevertheless, this method necessitates multiple rounds of interaction between the client and server and does not enable instant real deletion of data. To mitigate the risk of additional information leakage in SSE, forward and backward privacy concepts were informally introduced by Stefanov et al. [11]. Bost [24] has provided a formal definition of forward privacy, while Bost et al. [14] have defined formal backward privacy (Type-I, Type-II, and Type-III). Lately also, different DSSE schemes with varying features of update and privacy have been put forth in the literature [9], [10], [25]. However, it is noteworthy that these mentioned DSSE schemes support updating a pair of keyword and ID. To delete the data of one particular ID, different tokens for the keywords are generated and then sent to the server.

There are also some recent schemes to provide privacy and security of genomic data when queries are performed on this type of dataset, including [32], [33], [34] that utilised searchable encryption. However, they have not considered dynamic consent in their schemes.

## 2 Preliminaries

This section presents the necessary preliminaries. In general terms, we define an algorithm Alg to be efficient if it operates within probabilistic polynomial time. Additionally, we characterize a function fun(*λ*) as negligible, represented by negl(λ) if for any constant c>0, there is a threshold $\lambda_{0}$ in such a way that f(*λ*)$<1/\lambda^{c}$ is true for any $\lambda >\lambda_{0}$.

**Genomic data representation:** An organism's genetic blueprint is encapsulated within its genome. Comprising two intertwined strands of deoxyribonucleic acid (DNA), this genetic material encodes vital information in humans and numerous other species. Adenine, Guanine, Cytosine, and Thymine, abbreviated as A, G, C, and T, respectively, compose the fundamental components referred to as nucleotides. Human genomes consist of around 3 billion of these base pairs. Single nucleotide polymorphisms (SNPs) denote genetic variations where more than one base (A, G, C, or T) occurs within a population. Typically, SNPs manifest as biallelic, presenting only two potential variants (alleles). An individual's genetic makeup, or genotype, reflects the specific combination of alleles they possess. SNPs contribute significantly to the genetic diversity underlying various human traits, including factors such as susceptibility to diseases, commonly referred to as phenotypes. In this article, search on genomic database includes searching for a keyword that can be SNP, phenotype, or other information about data owners such as their gender or ethnicity.

**Symmetric Key Encryption:** A symmetric key encryption (SE) system comprises the following pair of algorithms, denoted as SE =(SE⋅ Encryption, SE⋅Decryption):

– ci←SE⋅Encryption(sk,m): When provided with sk∈SK as secret key and m∈M as message, this algorithm produces ci∈CI as ciphertext, where SK, M, and CI denote the key set, message set, and ciphertext set, respectively.
– m←SE⋅Decryption(sk,ci): With the secret key sk and the ciphertext *ci* as input, this algorithm retrieves the original message *m*.

Correctness. An SE scheme is deemed perfectly correct if, for every message m∈M and secret key sk∈SK, and for any ci←SE⋅Encryption(sk,m), it ensures that Pr[SE⋅Decryption(sk,ci)]=1.

Definition 1 We define an SE as being IND-CPA secure if, for every probabilistic polynomial time (PPT) adversary A, its advantage ${\mathrm{Adv}}_{\mathrm{SE},A}^{\mathrm{IND}-\mathrm{CPA}}(\lambda )=|\mathrm{Pr}\;\left[A\left(\mathrm{SE}\cdot \mathrm{Encryption}\;\left(sk,m_{0}\right)\right)=1\right]-\mathrm{Pr}[A\left(\mathrm{SE}\cdot \mathrm{Encryption}\;\left(sk,m_{1}\right)=1\right]|$ is negligible. Here, the secret key sk∈SK is kept confidential, and A selects $m_{0},m_{1}\in M$ of equal length. Furthermore, A is permitted to adaptively generate a polynomial number of encryption queries. For each message *m* in the message set M, the challenger responds with ci←SE⋅Encryption(sk,m).

**Searchable Symmetric Encryption (SSE):** Using traditional data encryption to address concerns about outsourced data security seems promising. However, it becomes complicated in practice because cloud servers cannot search encrypted data directly. This means users have to download and decrypt all data before searching, which is time-consuming. Searchable Encryption offers a solution by allowing data to be stored in encrypted form in the cloud while still enabling server searches. Searchable ciphertexts and search tokens are created by the secret key holder.

**Dynamic Searchable Symmetric Encryption (DSSE):** The DSSE scheme encompasses three protocols: Setup, Update, and Search. Their functionalities are outlined as follows:

– Protocol Setup(λ): The client initiates her secret key *K*, along with an empty state set *σ* with the security parameter *λ*. Subsequently, the client sends an empty encrypted database, denoted as EDB, to the server. Both the key *K* and state-set *σ* are kept confidential by the client.
– Protocol Update(K,σ,op,(w,id),EDB): Based on the parameter op∈add,del, the client either adds a new pair of keyword and file identifier (w,id) to the server or deletes a currently stored entry. Using the key *K* and state-set *σ*, the client transmits a new ciphertext of the entry (w,id) to the server if op=add. Otherwise (if op=del), she sends a delete token of the entry (w,id). Upon reception of the message, the server proceeds to update its database EDB accordingly.
– Protocol Search(K,σ,w,EDB): Given the key *K* and the state-set *σ*, the client transmits a search trapdoor associated with the keyword w to the server. The server searches for the keyword within EDB and supplies the client with all legitimate file identifiers.

DSSE correctness: In a DSSE scheme, it is essential that the system consistently retrieves all valid file identifiers.

When evaluating the security of DSSE, a common approach involves comparing the indistinguishability of a real game with that of an ideal game. The adversary is permitted to perform Update and Search queries in these games. In the real game, all entries and secret keys are authentic, and both the Update and Search protocols are correctly executed. Conversely, in the ideal game, the responses to all adversary queries are generated by a simulator utilizing only leakage functions. We posit that DSSE can be deemed secure if a simulator can effectively replicate an ideal game that cannot be distinguished from the real game.

Definition 2 (DSSE Adaptive Security). With leakage functions $L=(L^{\text{Setup}},L^{\text{Update}},L^{\text{Search}})$, a DSSE scheme D is deemed L-adaptively secure if, for any sufficiently large security parameter *λ* and adversary A, there exists an efficient simulator Sim=(Sim.Setup, Sim.Update, Sim.Search) such that $\left|\mathrm{Pr}\;\left[\mathrm{Real}\;A^{D}(\lambda )=1\right]-\mathrm{Pr}\;\left[\text{Ideal}A,Sim,L^{D}(\lambda )=1\right]\right|$ is negligible in *λ*, where the games are defined as follows:

– Real${}_{A}^{D}(\lambda )$: The real game represents the actual execution of DSSE protocols. Adversary A can issue queries of Update and Search adaptively with inputs (op,(w,id)) and w, respectively, and get the real transcripts generated by the DSSE protocol. Ultimately, adversary A outputs a bit.
– Ideal${}_{A,Sim,L}^{D}(\lambda )$: The simulator Sim emulates all transcripts. Adversary A can make identical queries as those in the real game. Sim utilizes the leakage functions L as input to simulate the corresponding records. Eventually, A outputs a bit.

Let Q denote a list of all queries (Update and Search), and each entry in Q has the format (u,op,(w,id)) for Update and (u,w) for Search queries. Here, u represents the query execution timestamp. Given a keyword w, the function sp(w) returns all timestamps of the Search queries on keyword w. Additionally, the function TimeDB(w) returns the undeleted file identifiers associated with keyword w along with their historical timestamps of addition. Furthermore, the function DelHist(w) provides the historical timestamps of all paired Add and Delete operations concerning keyword w. Below are the formal definitions of the aforementioned three functions.

sp(w)={u|(u,w)∈Q}

$\mathrm{TimeDB}(w)=\{(u,\mathrm{id})|(u,\;\text{add},(w,\mathrm{id}))\in Q\&\forall u^{\prime },\left(u^{\prime },\mathrm{del},(w,\mathrm{id})\right)\notin Q\}$

$\mathrm{DelHist}(w)=\{\left(u^{\mathrm{add}},u^{\mathrm{del}}\right)|\exists \mathrm{id},\left(u^{\mathrm{add}},\mathrm{add},(w,\mathrm{id})\right)\in Q\&\left(u^{\text{del}\;},\mathrm{del},(w,\mathrm{id})\right)\in Q\}$

With the above functions, forward and Type-III-backward privacy are defined in Appendix 1. We introduce our new backward privacy notion IDBP, suitable for SSE with ID-based updates like our ACE construction, in Section 5.

**Pseudorandom Function (PRF):** To encrypt search queries and tokens deterministically in our architecture, we employ PRFs. A pseudorandom function (PRF) [15] is a set of efficient functions where distinguishing between a randomly chosen function from the PRF family and a random oracle (a function producing outputs entirely at random) with significant advantage is computationally infeasible. These functions are fundamental in constructing cryptographic primitives and are defined as follows:

Let *F*1 and *F*2 be sets, $Fun:0,1^{\lambda }\times F1\to F2$ be a function, and $s\overset{\$}{\leftarrow }S$ represent the operation of randomly selecting an element from set *S*. Furthermore, Fun(F1,F2) denotes the set of all functions from *F*1 to *F*2, and *λ* signifies the security parameter for PRF. We consider *Fun* to be a pseudorandom function (PRF) if, for all efficient adversaries A, the advantage ${\mathrm{Adv}}_{Fun,A}^{\mathrm{prf}}(\lambda )=\mathrm{Pr}[A^{Fun(K,\cdot )}(\lambda )=1]-\mathrm{Pr}[A^{fun(\cdot )}(\lambda )=1]\leq \mathrm{negl}(\lambda )$, where the probability is determined by the randomness of A, $K\overset{\$}{\leftarrow }0,1^{\lambda }$, and $fun\overset{\$}{\leftarrow }\mathrm{Fun}(F1,F2)$.

**Trapdoor Permutations:** A trapdoor permutation *π* is defined as a permutation over a set D such that *π* can be effortlessly computed using a public key (PK), while the efficient computation of its inverse, $\pi^{-1}$, necessitates the utilization of a secret key (SK).

Formally, *π* is characterized as a trapdoor permutation with the key generation algorithm KG if, for every efficient adversary A, ${\mathrm{Adv}}_{\pi ,A}^{\mathrm{ow}}(\lambda )\leq \mathrm{negl}(\lambda )$ where

$${\mathrm{Adv}}_{\pi ,A}^{\mathrm{ow}}(\lambda )=\mathrm{Pr}[y\overset{\$}{\leftarrow }M,(\mathrm{SK},\mathrm{PK})\leftarrow \mathrm{KG}\;\left(\lambda \right),x\leftarrow A\left(\lambda ,\mathrm{PK},y\right):\pi_{\mathrm{PK}}(x)=y]$$

(*π* is one-way) implying that for every x∈D, $\pi_{\mathrm{PK}}\left(\pi_{\mathrm{SK}}^{-1}(x)\right)=x\text{ and }\pi_{\mathrm{SK}}^{-1}\left(\pi_{\mathrm{PK}}(x)\right)=x$ and $\pi_{\mathrm{PK}}(.)$ and $\pi_{\mathrm{SK}}^{-1}(.)$ is computed in polynomial time.

## 3 System model

This section presents the system model that shows the entities comprising the system and elucidates their respective roles and responsibilities. It also presents the threat model that establishes the assumptions pertaining to the adversary.

### 3.1 System model overview

**Figure 1:**
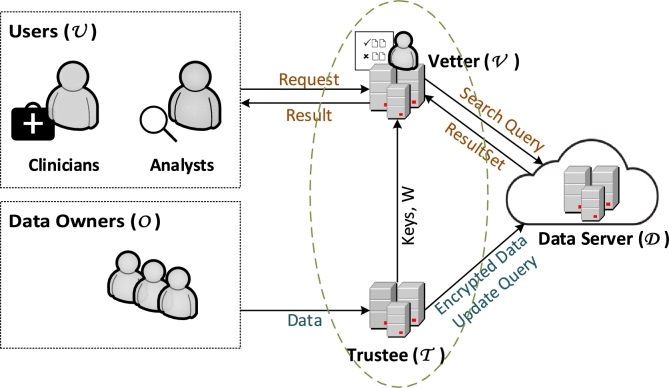
System design overview of ACE.

The envisioned model comprises several components (depicted in Fig. 1), including the data owner, data server (genomic sequence data database), data provider (trustee), and users (analysts or clinicians). Each component has specific roles as outlined below:

**Data owner (***O***):** An individual whose data is collected is denoted as a data owner. When a data owner visits a medical facility, such as a gene trustee, either as a patient or a study participant, their data is gathered and recorded. During this process, the data owner grants consent to the trustee to utilize their genetic data for additional studies or treatments. By notifying the trustee, the data owner can subsequently revoke the consent.

**Data provider or Trustee (**T**):** In our model, a medical institution, e.g., gene trustees, functions as data provider. The T entity maintains a registry of collected genomic data along with associated consent information. We presume the data provider to be trustworthy. The primary responsibilities of it include: encoding genomic data sequences, encryption of encoded sequences, and management of cryptographic keys. Moreover, T is able to insert new genomic data when new data owners provide their samples. T is also responsible for removing the genomic data of data owners who revoke their consent. This way the dynamic consent feature is embedded into the system and allows for the revocation to happen once the request is provided.

**Vetter (**V**):** In addition to the data provider, another trusted entity is depicted in Fig. 1 as a separate entity, which can also be merged with trustee. This entity receives keys from the trustee for the search phase, handles queries from users, and generates search tokens.

**Users (**U**):** Users transmit detailed queries to the trusted entity and await the outcome of query execution.

**Data Server (**D**):** The data server, denoted as D, maintains records of genomic data sequences. It carries out encrypted queries over encrypted data and sends the results back to the users. It also stores the newly inserted encrypted data from T and deletes the requested data based on received update queries from the T.

### 3.2 Threat model

The Data Server (D) should not be able to learn anything regarding the shared genomic data or the unencrypted query results that the analysts or clinicians run. This is our ideal security goal. The Data Server is an honest-but-curious (semi-honest) adversary. This demonstrates that D faithfully follows the protocol and harbors no intention of deliberately obtaining incorrect outcomes. However, D may seek to acquire additional information beyond the expected scope during or after the protocol execution. We consider the trustee as a trusted entity. Users (analysts or clinicians) may be unauthorized, thus they are authorized by the trustee, a trusted entity responsible for verifying the validity of queries. Lastly, we assume that D and U do not collude with each other. The discussion on the security model and analysis are given in Section 5.

Outsourcing data to the cloud introduces the risk of unauthorised access to sensitive information, posing a potential threat of data breaches and compromising confidentiality. This scenario creates the possibility of exposing sensitive data to interception or theft, thereby risking the abuse of confidential information. In our study, we prioritise both efficient functionality (identified requirements in Table 1) and security in handling data. We focus on securely storing information to prevent unauthorised disclosure of data. At the same time, our system aims to add/delete/retrieve specific data with minimal exposure to the server. By combining robust security measures and functionality, our approach seeks a harmonious balance. This strategy safeguards confidential information but also ensures smooth and controlled access, reducing the risk of unauthorised disclosure and access. Our commitment to both aspects, namely efficient functionality, and security, shapes the core of our methodology, aligning with the broader objective of delivering a comprehensive solution for effective genomic data storage on clouds with fast retrieval as well as addition and deletion properties.

## 4 ACE construction

To construct ACE, we considered the following main ideas that enable ACE to efficiently handle encrypted genomic data searches while providing robust data privacy and compliance with regulatory requirements.

– **Counter-Based Searchable Ciphertexts:** Our approach uses a counter-based design to create searchable ciphertexts efficiently. By traversing valid counter values, the server can locate matching ciphertexts for a given keyword with sub-linear search complexity. This reduces the need to go through all indices and improves search performance significantly.
– **Efficient Instant Physical Deletion based on ID:** To achieve secure data deletion when participants revoke consent, we store deltas (Δ) for each ID and issue a deletion token. This token allows the server to generate all indices related to the ID to be deleted, minimizing communication costs without the need for multiple tokens.
– **ID-Based Forward and Backward Privacy:** ACE ensures ID-based forward privacy by using trapdoor permutation (ST) to prevent new insertions from being linked to previous search tokens. For ID-based backward privacy, all IDs are encrypted to prevent the server from learning about deleted IDs, thereby preserving privacy during search queries.

The notations presented in Table 2 are used in the subsequent discussions.

**Table 2:** Notations.

Notation	Description
ID	Data Owner's unique ID
${\mathrm{ID}}^{\prime }$	Encrypted Data Owner's ID
w	A keyword
GDB(w)	The set of Data Owner IDs that contain that particular w
$W_{\mathrm{ID}}$	The set of keywords the data owner (with ID) has
GDB	Genomic DataBase; a set of $\left\{{\mathrm{ID}}_{i},W_{{\mathrm{ID}}_{i}}\right\}$
EGDB	Encrypted Genomic DataBase

### 4.1 Construction

1) Setup(λ): This algorithm is presented in Algorithm 1. This is the setup phase which is related to the key generation part and creating empty maps to be filled later with the added data. The Trustee T executes this algorithm. Given the security parameter *λ* as input, T runs this algorithm and outputs the empty map and dictionary EGDB= {EGDB1, EGDB2}, an empty map W[w], and a set of keys K. It selects random keys $K_{S},K_{1}$ for PRF *F* and $K_{T},K_{2}$ for PRF $F_{p}$ and the generator $g\overset{\$}{\leftarrow }G$. It also generates a set of (SK,PK) for *π* using the KeyGen algorithm of the trapdoor permutation. The EGDB1 stores deltas (that are used for generating tokens for deletion) for each ID, and the EGDB2 dictionary contains searchable ciphertexts in a counter-based design with the encrypted IDs. The EGDB is stored on the D, and the corresponding keys (for search and retrieval) along with a map **W** are provided to the V to generate search tokens. T retains all the keys for the update phases.

**Algorithm 1:**
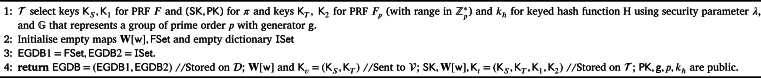
ACE.Setup.

2) $\mathrm{Update}(\{{\mathrm{ID}}_{i},W_{{\mathrm{ID}}_{i}}\}$, op=add,X) or Update(ID, op=del,X), where $X=\{\mathrm{SK},W[w],K_{t}=(K_{S},K_{T},K_{1},K_{2})$ that are stored on T, EGDB}: Based on the operation, op, needed to be performed, either add or delete an ID with its corresponding keywords ($\mathrm{ID},W_{\mathrm{ID}}$), different steps take place by Trustee T. In ACE, the term update-add means the data of several new Data Owners are provided to the Trustee (batch insertion), while update-del means a Data Owner revokes their consent and requests the removal of their data.

In ACE's batch insertion process, when adding a set of IDs with relevant keywords, the system retrieves the relative counters from the **W** map. If a counter is empty, a random element for $ST_{0}$ is chosen. For every w in the dataset, a tag and a key for encrypting the ID are generated. For all the IDs that have the keyword w an index $r_{\mathrm{ID}}$ and a tag ${\mathrm{tag}}_{\mathrm{ID}}$ are generated. To generate the dictionary which has the counter-based search capability ${\mathrm{ST}}_{c}$ is used, that acts as a counter. In the pseudo code, *π* is a trapdoor permutation and ${\mathrm{ST}}_{c}$ can be generated by using the secret key of the trapdoor permutation and ${\mathrm{ST}}_{c-1}$. Then, an index *ℓ* which is based on counter (${\mathrm{ST}}_{c}$ mod *p*) and w ($\mathrm{ta}g_{w}$) is generated and the relevant encrypted ID is appended to the dictionary with index *ℓ*. We use mod *p* to be able to perform the operation in group G of prime order *p*. These indices *ℓ* and the corresponding encrypted IDs create the ISet that is considered as the EGDB2. EGDB1 is a map that stores different deltas, Δ, for a particular ID. In this case, when looking for an ID, the corresponding deltas will be retrieved which are based on counter (${\mathrm{ST}}_{c}$), w ($\mathrm{ta}g_{w}$), ID ($\mathrm{ta}g_{\mathrm{ID}}$). This way, when a search token is sent to the server, it would not be able to calculate the indices in ISet using deltas and find the correlation of deltas and indices in the ISet. On the Vetter V and Trustee T sides, the map **W** associates each inserted keyword with its current ${\mathrm{ST}}_{c}$ and a counter *c*. Whenever a new document matching w is inserted, the entry W[w] is incremented. So, FSet and ISet are computed and stored on the data server and new ST and counter *c* are stored in **W**. An example of stored data in ACE is given in Appendix 2.

For deleting an ID when the consent is revoked, a tag for that particular ID is generated by Trustee T and sent to the Server D. Accordingly, the D retrieve the deltas in FSet and starts computing the corresponding indices in the ISet using deltas and the received token. After computation and searching for these indices, all the relative entries in FSet,ISet are removed by the D.

3) $\mathrm{Search}(W[w],K_{v},w$, $E{\mathrm{GDB}}_{2})$: The Vetter V generates a token for the search and also retrieves the corresponding counter and ST from map **W** to send to the D for the search process. The D starts computing the indices in the ISet based on the counter (using trapdoor permutation and its public key) and retrieves the encrypted IDs. The entire process is outlined in Algorithm 3. The D initializes an empty set RSet to store the encrypted IDs (${\mathrm{ID}}^{\prime }$) that match the query. Subsequently, the V receives the RSet and generates the key for decrypting the retrieved ${\mathrm{ID}}^{\prime }\in \mathrm{RSet}$ using $K_{v}$ and w (where Dec is the decryption algorithm).

**Algorithm 3:**
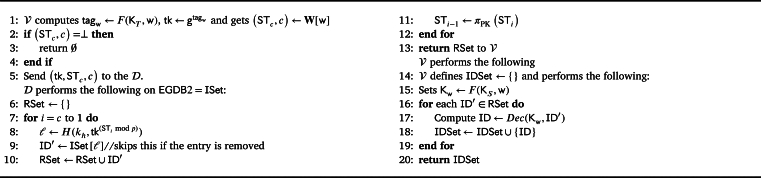
ACE.Search.

**Algorithm 2:**
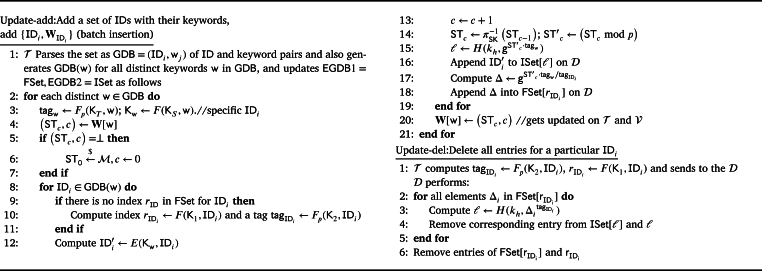
ACE.Update.

## 5 Security analysis

The SSE scheme's confidentiality definition relies on comparing real-world and ideal-world scenarios, with a leakage function determining what information the protocol exposes to adversaries. This setup involves two games: Real(λ), using our scheme, and Ideal(λ), simulated with our scheme's leakage. The leakage function, denoted by $L=\left(L^{Stp},L^{Srch},L^{Updt}\right)$, specifies what data is accessible to the adversary A. If A cannot distinguish between these scenarios, it suggests that the leakage stays within the predefined bounds of the leakage function.

To enable us to handle ID-based deletion queries in our security reduction of ACE, we define a non-adaptive security model where some information about the adversary's queries are defined by adversary in the beginning of the game using a data structure called query-info. We define query-info to be a set of queries defined by adversary in advance. This set includes: IDs to be deleted, keywords of those IDs to be searched before deletion (from this information, a set called $S_{i}\text{ with }\{t_{Srch}<t_{Del}\}$ for each ID_*i*_ is created that includes the keywords of that ID that are searched before being deleted). The update-add queries are not included in query-info if added IDs are not in the to-be-deleted list of IDs.

query-info$=\{({\mathrm{ID}}_{i},S_{i})|{\mathrm{ID}}_{i}\text{ will be deleted}\;\&\;S_{i}=\text{set of }w\in W_{{\mathrm{ID}}_{i}}|t_{Srch}<t_{Del}\}$

The formal definitions of the games are as follows:

- ${\mathrm{Real}}_{A}^{\Sigma }(\lambda )$: Given a dataset and query-info chosen by the adversary A, this game outputs EGDB using the real algorithms (Setup, Update-add) to A. The adversary can execute search and update-del queries within the provided query information, along with other search and update-add queries. The game returns the results obtained by executing Search and Update to A, after which A outputs a bit.

- ${\mathrm{Ideal}}_{A,S}^{\Sigma }(\lambda )$: When presented with a dataset and query-info chosen by A, this game utilizes a simulator $Sim\left(L^{Stp},L^{Updt}\right)$ to generate EGDB for the adversary A. Subsequently, it emulates the outcomes of the search query using the leakage function $Sim\left(L^{\text{Srch }}\right)$ and employs $Sim\left(L^{Updt}\right)$ to simulate the results for update (add or delete) queries. Moreover, it integrates the predefined query information (specified beforehand by A) while simulating the results for add queries. Finally, A produces a bit as output.

Definition 3 (Security with respect to the Server). The protocol P achieves semantic security against non-adaptive attacks with leakage L if, for all probabilistic polynomial time (PPT) adversaries A, there exists a PPT simulator Sim so that the absolute difference between the probabilities $\left|\mathrm{Pr}\;\left[{\mathrm{Real}}_{A}^{P}(\lambda )=1\right]-\mathrm{Pr}\;\left[{\mathrm{Ideal}}_{A,Sim}^{P}(\lambda )\right]\right|$ is negligible, denoted by negl(λ), where *λ* represents the security parameter.

We can demonstrate the security of our scheme within the Random Oracle Model, where we analyze the security of the construction assuming that H is represented as a random oracle.

### 5.1 Security assumptions

In this section, we define a new hard problem, D-ACE, that facilitates the proof of our theorem. We formally prove that D-ACE is a hard problem. Otherwise, DDH problem can be solved (a reduction from DDH to D-ACE is presented).

Definition 4 (Multi-instance DDH problem). Let G denote a cyclic group of prime order *p*. The multi-instance Decisional Diffie-Hellman (DDH) problem involves distinguishing the ensemble ${\left\{(g,g^{r_{i}},g^{s_{j}},g^{r_{i}s_{j}})\right\}}_{i,j}$ from ${\left\{(g,g^{r_{i}},g^{s_{j}},g^{z_{i,j}})\right\}}_{i,j}$ with independent uniform $z_{i,j}$s, where i=1,…,m and j=1,…,n, for some m,n polynomial in security parameter *λ*, g∈G and $r_{i},s_{j},z_{i,j}\in Z_{p}$ are chosen uniformly at random. The multi-instance of DDH assumption is valid if for all PPT distinguisher D, its advantage ${\mathrm{Adv}}_{D,G}^{DDH}(\lambda )$ satisfies:

$$\mathrm{Pr}[D{\left(g,g^{r_{i}},g^{s_{j}},g^{r_{i}s_{j}}\right)}_{i,j}=1]-\mathrm{Pr}[D{\left(g,g^{r_{i}}g^{s_{j}},g^{z_{i,j}}\right)}_{i,j}=1]|\leq \mathrm{negl}(\lambda ).$$

It is well known (by a standard hybrid reduction) that the hardness of multi-instance DDH for m,n=poly(*λ*) is equivalent to the standard one-instance DDH problem with m=n=1 [16].

Definition 5 (D-ACE problem). Let G be a cyclic group of prime order *p*, and *π* be a permutation with a KeyGen algorithm that generates a set of key (PK,SK) for the *π* evaluation, *λ* be the security parameter, A be the adversary, and consider the game in Algorithm 4 that is played between an adversary A and a challenger and is parameterized by a bit v∈{0,1}. The adversary's distinguishing advantage is $\left|\mathrm{Pr}[v=v^{\prime }]-(1/2)\right|$ and we say that D-ACE assumption holds if for all PPT adversary, its distinguishing advantage $\left|\mathrm{Pr}[v=v^{\prime }]-(1/2)||\leq \mathrm{negl}(\lambda \right)$.

**Algorithm 4:**
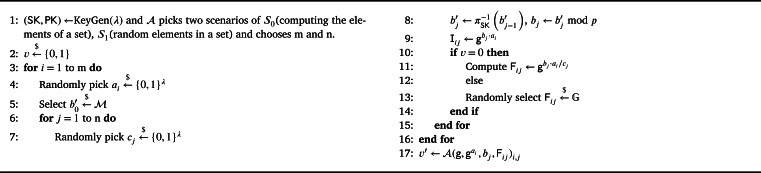
D-ACE.

Lemma 1 *If there exists an efficient algorithm*A*with a non-negligible advantage against D-ACE, then we can construct an efficient algorithm*D*with a non-negligible advantage against DDH.*

The proof is discussed in the Appendix 3.

### 5.2 Leakages

Consider Q as a collection comprising all Update and Search queries. Each entry within Q conforms to one of three formats: ($u,\mathrm{add},({\mathrm{ID}}_{1},{\mathrm{ID}}_{2},\ldots )$), (u,del,ID), or (u,w), representing Update (add), Update (delete), and Search queries, respectively, where u denotes the timestamp of query issuance. We define a function F of the inputs (ID,w) as a randomization function, that outputs a random element for each pair of (ID,w). We also define a function T of the input w as a randomization function, that outputs a random element for each w. The leakages' definitions are as follows.

– When adding several IDs and their relative keywords in a batch insertion, function $N_{\mathrm{ID}}$ returns the total number of IDs that have been added.
  $N_{\mathrm{ID}}(\mathrm{add})=\left\{\left(\text{Number of added }\mathrm{ID}\text{s in one batch insertion}\right)\right\}$
– When adding several IDs and their relative keywords in a batch insertion, function $NW_{\mathrm{ID}}$ returns the total number of ws that have been added for ID.
  $NW_{\mathrm{ID}}(\mathrm{add})=\{(\text{Number of added }w\text{s for a particular }\mathrm{ID}$ in a batch insertion)}
– Given an identifier ID, AddHist(ID) returns the history timestamp of Add operation about ID that has been added (batch insertion) with some other IDs.
  $\mathrm{AddHist}(\mathrm{ID})=\{\left(u^{\mathrm{add}}\right)|\exists \text{set of }\mathrm{ID}\text{s},\left(u^{\mathrm{add}},\mathrm{add},(\text{set of }\mathrm{ID}\text{s including }\mathrm{ID})\right)\in Q\}$
– For a given identifier ID, the function DelHist(ID) provides the historical timestamps of all paired Add and Delete operations related to ID.
  $\mathrm{DelHist}(\mathrm{ID})=\{\left(u^{\mathrm{add}},u^{\mathrm{del}}\right)|\exists \mathrm{ID},\left(u^{\mathrm{add}},\mathrm{add},(\text{set of }\mathrm{ID}\text{s including }\mathrm{ID})\right)\in Q$$\text{ and }\left(u^{\text{del}\;},\mathrm{del},\mathrm{ID}\right)\in Q\}$
– Given an identifier ID, function Delindex(ID) returns the correlation of stored deltas in FSet with search indices in ISet, that is revealed after deletion of ID.
  Delindex(ID)={Δ2ℓ: matching delta with search index after deleting ID}
– Given an identifier ID, function Delw(ID) returns a set of all $T_{i}\left(w\right)$ for all $w_{i}$ that have been deleted in $u^{\mathrm{del}}$ and have been searched in time $u_{i}<u^{\mathrm{del}}$. Otherwise, returns nothing. Note: this information can be derived from query-info and from the defined set of *S*.
  $\mathrm{Delw}(\mathrm{ID})={\left\{{\{T}_{i}(w_{\mathrm{ID}})\right\}}_{i}|\left(u^{\mathrm{del}},\mathrm{del},\mathrm{ID}\right)\in Q\text{ and }w_{i}\text{ searched before }u^{\mathrm{del}}\}$
– For a given keyword w, the function sp(w) retrieves all timestamps associated with the Search queries pertaining to the keyword w and rp(w) returns the timestamps and the randomized output related to the IDs returned in the search of w.
  sp(w)={u|(u,w)∈Q}, rp(w)={(u,F(ID,w))|(u,w)∈Q}
– Given a keyword w, TimeDB(w) returns all F outputs related to the undeleted identifiers (IDs) that possess keywords w and historical timestamps for adding these IDs.
  TimeDB(w)={(u,F(ID,w))|(u,add,(ID))∈Q$\text{ and }\forall u^{\prime },\left(u^{\prime },\mathrm{del},(\mathrm{ID})\right)\notin Q\}$
– Given a keyword w, skipped tokens returns all the search tokens for w that were deleted before the time of search for w.
  $\mathrm{skipped tokens}(w)=\{(u^{\mathrm{srch}},F(\mathrm{ID},w))|\left(u^{\mathrm{del}},\mathrm{del},\mathrm{ID}\right)\in Q\text{ and }\mathrm{ID}\text{ that has }w$$\text{ has been deleted before }u^{\mathrm{srch}}\}$
– Given an identifier ID, function BFF(ID) returns the set of entries (i.e., indices, deltas, encrypted IDs) that have been added in one batch insertion and have not been deleted yet.
  BFF(ID)={(set of entries related to IDs,w)|AddHist(IDs)=AddHist(ID)}

In this article, ID-based DSSE (IDDSSE) is considered as a dynamic SSE that offers updates based on the IDs; IDs with relevant keywords are added or deleted in the update phase. We define the below definitions of IDFP and IDBP.

Definition 6 An IDDSSE scheme exhibits ID-forward privacy if Update (add) queries refrain from disclosing the keywords associated with the IDs being updated. Only the count of IDs and the total number of keywords in a batch update are disclosed. Formally, IDFP states that a L-non-adaptively secure IDSSE scheme achieves ID-forward privacy iff the update leakage function $L^{\text{Updt-add}}$ can be expressed as:$L^{Updt-\mathrm{add}}(\mathrm{add},{\left\{{\mathrm{ID}}_{i},W_{{\mathrm{ID}}_{i}}\right\}}_{i})=L^{\prime }(\{\mathrm{add},NW_{{\mathrm{ID}}_{i}}(\mathrm{add}),\mathrm{AddHist}({\mathrm{ID}}_{i})\})$ where $L^{\prime }$ is stateless.

Definition 7 An IDDSSE scheme is ID-backward-private if it does not reveal the IDs that have already been deleted but it leaks if the search on being deleted w happened before deletion, the count of IDs currently associated with keyword *w*, along with their insertion timestamps, and the correspondence between deletion updates and batch insertions. Formally, IDBP defines that a L-non-adaptively secure IDSSE scheme achieves ID-backward privacy iff the search and update leakage functions $L^{\text{Srch }},L^{\text{Updt-del}}$ can be expressed as: $L^{Updt-\mathrm{del}}(\mathrm{del},\mathrm{ID})=L^{\prime }(\{\mathrm{del},\mathrm{Delw}(\mathrm{ID}),\mathrm{DelHist}(\mathrm{ID})\})$ and $L^{Srch}(w)=L^{\prime \prime }(\{\mathrm{sp}(w),\mathrm{rp}(w),\mathrm{TimeDB}(w)\})$ where $L^{\prime }$ and $L^{\prime \prime }$ are stateless.

Theorem 1 *Consider π as a one-way trapdoor permutation, F as a secure pseudorandom function (PRF), and (Enc, Dec) as a secure symmetric encryption scheme. Assuming that the D-ACE assumption holds in* G *, by defining the leakage function* L *as below, ACE is* L *-non-adaptively-secure and satisfies IDFP, IDBP.* $L^{Stp}(\lambda )=\{\lambda \}$ *,* $L^{Updt}(\mathrm{add},\{{\mathrm{ID}}_{1},W_{{\mathrm{ID}}_{1}}\},\{{\mathrm{ID}}_{2},W_{{\mathrm{ID}}_{2}}\},\ldots )=$ *,* {add *,* $N_{\mathrm{ID}}(\mathrm{add})$ *,* $NW_{\mathrm{ID}}(\mathrm{add})$ *,* AddHist(ID)} *,* $L^{Updt}(\mathrm{del},\mathrm{ID})=\{\mathrm{del}$ *,* Delw(ID) *,* DelHist(ID) *,* BFF(ID)} *, and* $L^{Srch}(w)=\{\mathrm{sp}(w)$ *,* rp(w) *,* TimeDB(w),skipped tokens}

Proof Sketch: In order to establish the security of our scheme, we develop a simulator that utilizes leakage functions as inputs to mimic the protocols Setup, Update, and Search. We illustrate that the simulated scheme cannot be distinguished from the real scheme under non-adaptive attacks. We construct multiple games based on the real-world game, each featuring incremental modifications. In Game G_1_, instead of using a pseudo-random function for generating tags, we pick a new random tag for each new keyword and ID. In G_2_, we encrypt a constant 0 using symmetric encryption encrypting IDs. In G_3_, we modify the Update phase to use random strings instead of hash function outputs. The simulator employs query-info to generate entries; for non-deleted, non-searched entries, it presents them as independent random. By distinguishing between games, we can deduce problems D-ACE, one-wayness of the trapdoor permutation. The simulator's advantage is 0 in final game. The complete formal proof is provided in the supplementary material.

Discussion: In our system model, T and V fulfill separate roles, as detailed in section 3. The U communicates with the V, which does not possess write privileges, ensuring a separation of privileges for enhanced security. This approach safeguards against compromise, as a breach in one entity does not affect the other. To address information leakages in secure searchable encryption (SSE) schemes, oblivious RAM (ORAM) techniques have been considered [17], [18]. However, ORAM introduces significant computational and bandwidth costs, making it impractical for efficient SSE. Thus, practical SSE schemes aim to strike a balance between information leakage and efficiency, achieving acceptable performance.

## 6 Analytical performance comparison

This section provides a performance evaluation comparing our ACE with other relevant methods from different perspectives. The detailed comparison is presented in Table 3.

– Update-Addition: When adding one ID (with its all relevant keywords) to the database, the computation that is needed and the communication complexity are in the order of the number of keywords the ID has for ACE, [13] and [12]. If we add n IDs with their keywords, the computation and communication complexity also increases by the number of IDs in ACE, [13] and [12].
– Update-Deletion: To delete an ID with the relevant keywords, the computation is in the order of the number of keywords for ACE, [13] and [12]. However, the communication complexity increases in proportion to the number of keywords for [13] and is a small token for ACE and [12].
– Search: Search computation complexity increases in proportion to the number of matched IDs in ACE, and it depends on the number of updates that has happened before search on w in [13]. In [12], the search complexity increases in proportion to the number of matched IDs for the keyword that is searched, and if a deletion happened before search, it needs to perform some computations to remove data in the search phase. However, ACE completes the update (addition and deletion) in their own phase and do not postpone any parts of update to the search phase.
– Storage: The storage requirement scales with the multiplication of the number of records and the number of keywords. In other words, it depends on the total count of (ID, w) pairs in the dataset for all three schemes listed in Table 3.

**Table 3:** Computational and communication costs.

Reference		ACE	[13]	[12]
Comp.	Addition	*x*(2*T*_*F*_ + 2*T*_*e*_ + *T*_*S*_ + *T*_*h*_)+2*T*_*F*_ + *T*_*E*_	*x*(2*T*_*F*_ + *T*_*h*_ + *T*_*X*_)+*T*_*E*_	*x*(3*T*_*F*_ + 3*T*_*h*_ + 3*T*_*X*_)+*T*_*F*_
	Deletion	2*T*_*F*_ + *x*(*T*_*e*_ + *T*_*h*_)	*x*(2*T*_*F*_ + *T*_*h*_ + *T*_*X*_)+*T*_*E*_	2*T*_*F*_ + *x*(2*T*_*h*_ + 3*T*_*X*_ + *T*_*R*_)
	Search	*T*_*F*_ + *α*(*T*_*e*_ + *T*_*h*_ + *T*_*S*_)	2*T*_*F*_ + *T*_*h*_ + *N*_*U*_⁎(*T*_*h*_ + *T*_*D*_)	2*T*_*F*_ + *α*(*T*_*h*_ + *T*_*X*_)+*N*_*D*_⁎(*T*_*h*_ + 3*T*_*X*_)

Stor.	Storage Size	*r*(*ℓ*_*F*_ + *x*(*ℓ*_*E*_ + *ℓ*_*D*_ + *ℓ*_*h*_))	*rx*(2*ℓ*_*h*_ + *ℓ*_*E*_)+*N*_*U*_⁎(2*ℓ*_*h*_ + *ℓ*_*E*_)	3*rx*(*ℓ*_*h*_ + *ℓ*_*F*_)

Comm.	Addition	*ℓ*_*F*_ + *x*(*ℓ*_*E*_ + *ℓ*_*D*_ + *ℓ*_*h*_)	*x*(2*ℓ*_*h*_ + *ℓ*_*E*_)	3*x*(*ℓ*_*F*_ + *ℓ*_*h*_)
	Deletion	2*ℓ*_*F*_	*x*(2*ℓ*_*h*_ + *ℓ*_*E*_)	2*ℓ*_*F*_
	Search	*ℓ*_*F*_ + *ℓ*_*D*_	*ℓ*_*F*_ + *ℓ*_*h*_	2*ℓ*_*F*_

**Notations**: $T_{F}$: Time taken for pseudorandom function (PRF) computation; $T_{e}$: Time taken for exponentiation computation; $T_{h}$: Time taken for hash function computation; $T_{E}$: Time required to encrypt a block using symmetric encryption; $T_{S}$: Time taken for trapdoor permutation computation; $T_{X}$: Time taken for XOR operation computation; $T_{R}$: Time required to overwrite an entry; $N_{U}$: Number of updates; $N_{D}$: Number of deletions; *α*: Number of records matching the searched keyword; *x*: Number of keywords associated with an ID; *r*: Number of records in the database; $\ell_{D}$: Size of an element within the Diffie-Hellman (DH) group; $\ell_{F}$: Length of the output from a pseudorandom function (PRF); $\ell_{E}$: Length of the block in symmetric encryption (SE); $\ell_{h}$: Size of the output produced by the hash function H.

This analytical comparison highlights the efficiency of ACE in terms of its search and update mechanisms. While both ACE and the other scheme in [12] offer deletion based on ID, ACE stands out by providing instant real deletion without any negative implications. Additionally, ACE ensures low communication costs for both search and deletion operations.

## 7 Experimental evaluations

We implemented ACE and evaluated it using different datasets on the following hardware and software configuration. The hardware configuration includes an Intel i7-11850H CPU and 64GB of memory, providing robust computational power for our cryptographic operations and data management tasks. The selection of the Fedora 35 x64 operating system is based on its compatibility with the chosen software components. We utilised Java 16 as the compiler for its efficiency and versatility in implementing cryptographic algorithms. The cryptographic library of choice is Bouncy Castle [19], renowned for its comprehensive set of cryptographic primitives and reliable implementation. We used the database Redis [20] for its renowned speed, low-latency responsiveness, and scalability. To further enhance security, we employed AES-128 based CMAC for PRF and SHA-512 based HMAC for PRF_*p*_, ensuring robust pseudo-random function generation [15], [21]. The implementation of the Trapdoor Permutation *π* [14] was accomplished using RSA-2048, a widely accepted standard in cryptographic protocols. Our code is available at https://drive.proton.me/urls/KZJMSC639G#HqHLc9xGCUp1.

The dataset used for testing ACE is a genomic dataset with SNP information, phenotypes, gender, and ethnicity of 69 data owners obtained from The Harvard Personal Genome Project (PGP) [22]. To expand our dataset for comprehensive testing, we also created synthetic datasets with varying numbers of records and keywords (from $5⁎10^{4}$ to $4⁎10^{6}$ (ID, w) pairs) to analyse ACE's performance. In our experiments, we prioritise reporting the time required for deletion, addition, and search operations. Additionally, we emphasise the size of the ciphertext added and the token for computing costs. These aspects are crucial for evaluating the efficiency and performance of our system in handling dynamic genomic data.

In the following sections, we delve into the Update-Addition, Update-Deletion, and Search evaluations. These evaluations are crucial components in understanding the system's real-time adaptability and responsiveness to dynamic changes in the dataset and also searchability. The Update-Addition examines the addition time of new data into the system and the ciphertext size that is added. The Update-Deletion assesses the system's performance in removing data and the token size that is used to delete data. By comprehensively examining these aspects, we gain insights into the system's ability in handling dynamic data modifications while providing search functionality.

**Figure 2:**
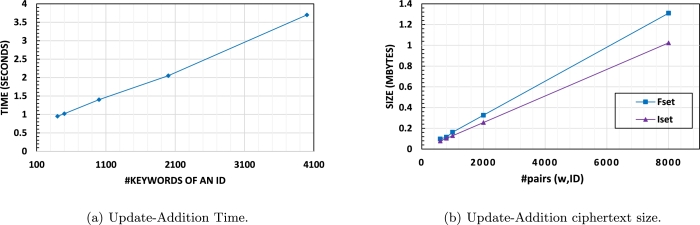
Update-Addition of 2 IDs with different number of keywords.

**Update-Addition:**Fig. 2 (a) shows the time cost for adding two IDs to the database in ACE, considering the number of keywords associated with the IDs. As ACE performs batch insertions, the number of keywords affects the addition time. On the other hand, the communication cost, depicted in Fig. 2 (b), is determined by the size of the encrypted data being added to the database. The ciphertext size increases with the number of (ID, w) pairs being added, represented as #pairs (w,ID) = 2⁎(#keywords) in this evaluation.

**Figure 3:**
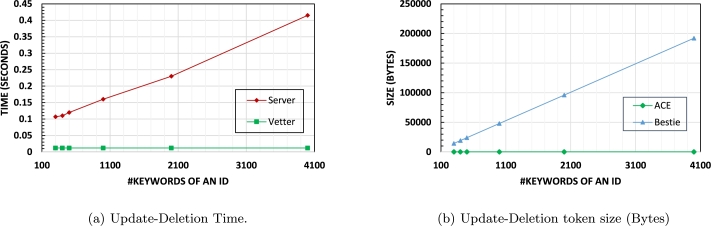
Update-Deletion of 1 ID with different number of keywords.

**Update-Deletion**: ACE's Update-del algorithm efficiently removes relevant data when consent is revoked or data needs to be deleted from the database. The deletion time remains constant, regardless of the number of keywords associated with a data owner (Fig. 3 (a)). Since ACE supports deletion based on an ID, the deletion token's size sent from vetter to server is constant, and the required bandwidth remains unaffected by the number of keywords for IDs (Fig. 3 (b)). In contrast, schemes that support deletion of (ID, w) pairs experience increased bandwidth requirements, as a new token must be generated and sent to the server for each keyword, resulting in linear growth (the token size for Bestie protocol in [13] is calculated from the sizes discussed in their paper).

**Figure 4:**
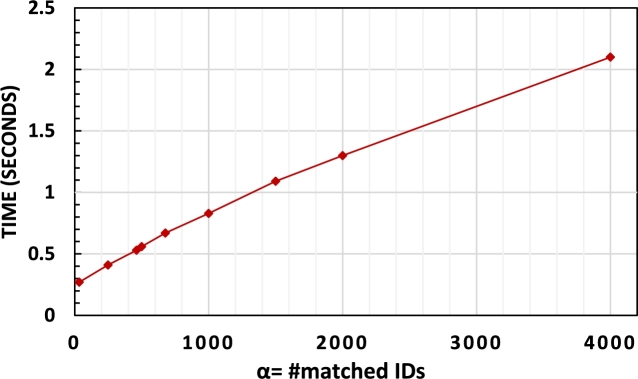
Total search time with different number of matched IDs.

**Search:** In comparison to [13] and [12], ACE achieves instant deletion, meaning data is deleted upon request without postponing or deferring the deletion process to the search phase. The search time in ACE increases with the number of matched IDs for the searched keyword (see Fig. 4). Additionally, there is an initialization time cost of approximately 200ms due to Java processing, which is considered in the presented search results.

ACE offers several advantages, including instant deletion with low communication complexity (one deletion token/non-interactive) and ID privacy (Table 1). Although ACE may not have the fastest search time (Fig. 4), it still demonstrates reasonable performance compared to earlier schemes lacking these features (See Table 4). For instance, ACE achieves a significant speedup in comparison to Janus++ and Janus, two well-known DSSE schemes that are evaluated in [9], with search times of 0.38s and 1.3s, respectively, when the number of matched IDs (*α*) is 200 and 2,000 (that is 10 times and 700 times speedup). Note that the performance of ACE in Java is slightly slower than C++ implementations [13] due to compiler differences. Table 4 compares different schemes and shows the search time with different number of matched IDs.

**Table 4:** Search time in different schemes with different number of matched IDs.

*α*^‡^	Scheme	Search time^†^
200	[12]^⁎^	400 ms
	[13]	<200 ms
	ACE	380 ms

2,000	[14]^⁎⁎^	700 s
	[9]^⁎⁎⁎^	10 s
	ACE	1.3 s

^‡^The comparison with different schemes is presented with different number of matched IDs since the results are extracted from the cited papers and they evaluated their schemes with different *α*s; ^†^: These are approximate times that are extracted from the schemes' provided graphs in their papers; ^⁎^: Data is extracted from their paper with $|\mathrm{DB}|=10^{7}$; ^⁎⁎^: Data is extracted from [9] with number of deletions=100 for the Janus protocol in this paper; ^⁎⁎⁎^: number of deletions=100.

**Storage:** The storage cost on server side (FSet, ISet), and on vetter and trustte sides (W) are presented in Table 5 with 1,000 number of IDs. The results are for different datasets with 1,000 number of IDs and different number of keywords. The storage size on the server side increases when the number of IDs or the number of keywords of an ID increases, but the size of W depends only on the number of distinct keywords.

**Table 5:** Storage size (original, encrypted FSet, ISet, and W).

#Keywords(x)	Original	FSet	ISet	W
1,000	5.7 MB	164 MB	137 MB	1.5 MB
4,000	26.2 MB	656 MB	546 MB	6 MB

## 8 Limitations

This section outlines the limitations of the study, focusing on challenges and underlying assumptions.

Although the proposed scheme provides effective privacy preservation, there is always room for improvement. In this regard, reducing the amount of information leaked which is defined by the leakage function or validating the scheme's security against adaptive attacks, thereby exploring a different security model can be considered as a future area of investigation. Furthermore, further research can be conducted to investigate the trade-off between privacy and utility, and how it can be optimised for different use cases. Moreover, diversifying the dataset used for experimentation, incorporating real-world genomic data with appropriate consent for research purposes instead of a combination of real-world and synthetic data and evaluating the applicability of the novel algorithms across different types of datasets represents an avenue for future research endeavors.

## 9 Conclusion

In this paper, we present ACE, a novel scheme that effectively tackles the challenges of consent revocation to provide physical deletion of data in a non-interactive instant manner based on data owners' identifiers (IDs). By implementing physical deletion of a data owner's information upon consent revocation, ACE ensures compliance with privacy regulations and offers robust privacy protection. It also provides all the required above-mentioned features efficiently while preserving privacy. We define and prove the hardness of the D-ACE problem, which underpins our security analysis. Additionally, we introduce two new privacy notions, ID-based forward privacy (IDFP) and ID-based backward privacy (IDBP), and use these tools to facilitate our formal security proof of ACE. Through our evaluations with real-life and synthetic genomic datasets, we demonstrate ACE's performance and applicability, while providing its advantage of IDFP/IDBP and instant ID-based deletion. Overall, ACE presents a comprehensive and efficient solution for securely searching encrypted genomic data, with practical implications extending to diverse application domains. This can be the applications that store data of data owners, add data in time, search for the keywords, and needs deletion of related data of a data owner once requested. This is an important area of research in privacy-preserving genomic data handling when considering consent revocation and our future work is to introduce hard problems to prove ACE achieves privacy under adaptive attacks.

## Ethics declarations

This study was reviewed and approved by 10.13039/501100001779Monash University Human Research Ethics Committee (MUHREC) and 10.13039/501100000943CSIRO Health and Medical Human Research Ethics Committee, with the approval numbers 27361 and 2021-005-RR, respectively. The real data extracted from the referenced source in this article has the consent of data owners for their data to be used in research purposes and the results be published.

## CRediT authorship contribution statement

**Sara Jafarbeiki:** Writing – original draft, Validation, Resources, Project administration, Methodology, Investigation, Formal analysis, Data curation, Conceptualization. **Amin Sakzad:** Writing – review & editing, Validation, Supervision, Resources, Methodology, Investigation, Formal analysis, Conceptualization. **Ron Steinfeld:** Writing – review & editing, Validation, Supervision, Methodology, Investigation, Formal analysis, Conceptualization. **Shabnam Kasra Kermanshahi:** Writing – review & editing, Validation, Supervision, Methodology, Investigation, Formal analysis, Conceptualization. **Chandra Thapa:** Writing – review & editing, Validation, Supervision, Investigation, Formal analysis. **Yuki Kume:** Writing – review & editing, Validation, Software, Resources.

## Declaration of Competing Interest

The authors declare that they have no known competing financial interests or personal relationships that could have appeared to influence the work reported in this paper.

## Figures and Tables

**Table 6:** Example of FSet and ISet in ACE assuming ${\mathrm{ID}}_{1}$ has keywords w1,w2, ${\mathrm{ID}}_{2}$ has keywords w1,w3, and ${\mathrm{ID}}_{3}$ has keywords w1,w3.

FSet	
$r_{{\mathrm{ID}}_{1}}$	$\Delta_{11}=g^{{\mathrm{ST}}_{c_{1}}\cdot {\mathrm{tag}}_{w_{1}}/{\mathrm{tag}}_{{\mathrm{ID}}_{1}}}$, $\Delta_{12}=g^{{\mathrm{ST}}_{c_{2}}\cdot {\mathrm{tag}}_{w_{2}}/{\mathrm{tag}}_{{\mathrm{ID}}_{1}}}$
$r_{{\mathrm{ID}}_{2}}$	$\Delta_{21}=g^{{\mathrm{ST}}_{c_{1}+1}\cdot {\mathrm{tag}}_{w_{1}}/{\mathrm{tag}}_{{\mathrm{ID}}_{2}}}$, $\Delta_{23}=g^{{\mathrm{ST}}_{c_{3}}\cdot {\mathrm{tag}}_{w_{3}}/{\mathrm{tag}}_{{\mathrm{ID}}_{2}}}$
$r_{{\mathrm{ID}}_{3}}$	$\Delta_{31}=g^{{\mathrm{ST}}_{c_{1}+2}\cdot {\mathrm{tag}}_{w_{1}}/{\mathrm{tag}}_{{\mathrm{ID}}_{3}}}$, $\Delta_{33}=g^{{\mathrm{ST}}_{c_{3}+1}\cdot {\mathrm{tag}}_{w_{3}}/{\mathrm{tag}}_{{\mathrm{ID}}_{3}}}$



ISet	
$H(k_{h},g^{{\mathrm{ST}}_{c_{1}}\cdot {\mathrm{tag}}_{w_{1}}})$	$E(K_{w_{1}},{\mathrm{ID}}_{1})$
$H(k_{h},g^{{\mathrm{ST}}_{c_{2}}\cdot {\mathrm{tag}}_{w_{2}}})$	$E(K_{w_{2}},{\mathrm{ID}}_{1})$
$H(k_{h},g^{{\mathrm{ST}}_{c_{1}+1}\cdot {\mathrm{tag}}_{w_{1}}})$	$E(K_{w_{1}},{\mathrm{ID}}_{2})$
$H(k_{h},g^{{\mathrm{ST}}_{c_{3}}\cdot {\mathrm{tag}}_{w_{3}}})$	$E(K_{w_{3}},{\mathrm{ID}}_{2})$
$H(k_{h},g^{{\mathrm{ST}}_{c_{1}+2}\cdot {\mathrm{tag}}_{w_{1}}})$	$E(K_{w_{1}},{\mathrm{ID}}_{3})$
$H(k_{h},g^{{\mathrm{ST}}_{c_{3}+1}\cdot {\mathrm{tag}}_{w_{3}}})$	$E(K_{w_{3}},{\mathrm{ID}}_{3})$

**Algorithm 5:**
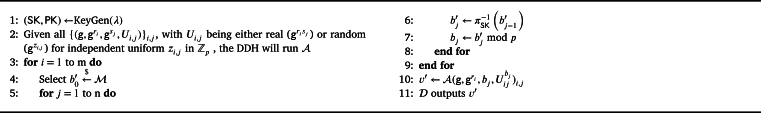
Reduction from DDH to D-ACE.

**Figure 5:**
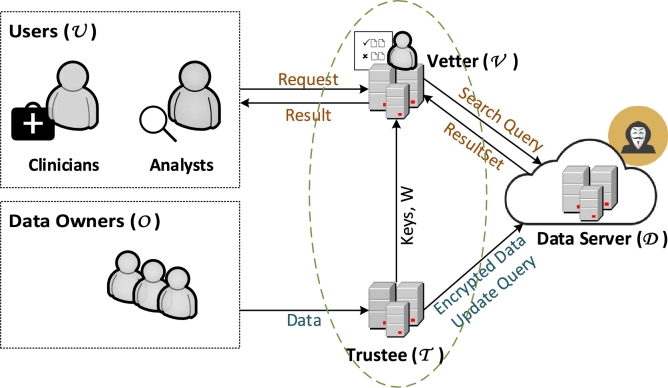
Threat model.

**Table 7:** Data representation with SNPs.

Data owner ID	SNP_1_	SNP_2_	SNP_3_	SNP_4_	...	Phenotype	Gender	Ethnicity
1	AA	CT	CT	AA	…	Cancer B	Male	Black
2	AG	TT	TT	AA	…	Cancer A	Female	Black
3	AA	TT	CT	AG	…	Cancer B	Male	White

## Data Availability

The data linked to this study has not been deposited into a publicly accessible repository. The actual data is obtained from The Harvard Personal Genome Project (PGP) and is referenced within this article [22], and the comprehensive dataset, comprising both the real and synthetic components, is not shared. Permission to use the dataset for research purposes and to publish the results has been obtained from the ethics committees of our respective institutions. We uphold the principles of ethical data usage and privacy, respecting the permissions granted for the specific research context outlined in this study.

## Appendix A

**Appendix** 1 **: Background**

(Forward and Type-III-Backward privacy). A DSSE scheme Σ achieving L-adaptive security is considered forward-and-Type-III-backward private if the leakage functions $L^{\text{Update}}$ and $L^{\text{Search}}$ associated with Update and Search operations satisfy the following condition:

$L^{\text{Update}}(\mathrm{op},w,\mathrm{id})=L^{\prime }(\mathrm{op})$ and

$L^{\text{Search}}(w)=L^{\prime \prime }(\mathrm{sp}(w),\mathrm{TimeDB}(w),\mathrm{DelHist}(w))$ where $L^{\prime }$ and $L^{\prime \prime }$ are stateless.

There are two further types of backwards privacy, referred to as Type-I and Type-II backward privacy, in addition to Type-III backward privacy. A search query only reveals the total number of updating w and TimeDB(w) in order to maintain Type-I backwards privacy. The timestamps of updating w, however, can also be leaked by a Search query when Type-II backward privacy is used. The Type-III backward privacy has been defined as an example and the precise formulations of Type-I and Type-II backward privacy can be located in [14].

**Appendix** 2 **: An Example of Stored Data in ACE**

Table 6 shows an example of the stored data on data server. Stored deltas in FSet where $\Delta_{ij}$ (*i* determines the ${\mathrm{ID}}_{i}$ and *j* determines $w_{j}$) help with the deletion of an ID's data without revealing any relationship between the entries of FSet and ISet before deletion. The deltas are generated using ST acting as counters (they provide privacy features that are discussed in details in section 5), $\mathrm{ta}g_{w}$ related to the keyword $w_{j}$ and $\mathrm{ta}g_{\mathrm{ID}}$ related to the ${\mathrm{ID}}_{i}$. The indices in ISet can be generated using deltas and $\mathrm{ta}g_{\mathrm{ID}}$. This relationship between entries in FSet and ISet is not computable by server; unless a deletion of ID needs to happen.

**Appendix** 3 **: Proof of** Lemma 1

The reduction algorithm (Algorithm 5) uses the $r_{i}$, $s_{j}$ of the DDH input instance as the $a_{i}$, $1/c_{j}$ of the D-ACE instance, respectively, whereas the $b_{j}$s of the D-ACE instance are simulated by D itself exactly as in the D-ACE game. The reduction can be analyzed as follows by considering the two possible cases of inputs to D. If the input to algorithm D comes from the real DDH distribution i.e., $U_{i,j}=g^{r_{i}s_{j}}$, then the last input to A in line 10 is $U_{i,j}^{b_{j}}=g^{r_{i}s_{j}b_{j}}=g^{a_{i}b_{j}/c_{j}}$, exactly as in the D-ACE real game (v=0), while if the input to D comes from the random DDH distribution i.e., $U_{i,j}=g^{z_{i,j}}$, then the last input to A in line 10 is $U_{i,j}^{b_{j}}=g^{z_{i,j}b_{j}}$, which are uniform and independent group elements if $b_{j}\neq 0$ for all *j*. Therefore, adv(D) can differ from adv(A) by at most the probability of the event B that one of the $b_{j}$s =0.

In Algorithm 5, when $z_{ij}$ is uniform, we want $z_{ij}\cdot b_{j}$ to be uniform. If $b_{j}$ is invertible in mod *p*, uniform $z_{ij}$ mod *p* gives uniform $z_{ij}\cdot b_{j}$ mod *p*. Based on line 4 in Algorithm 5, $b_{j}^{\prime }$ is uniform in M since $b_{0}^{\prime }$ is uniform and $b_{j}^{\prime }$ gets mapped through an iterated permutation (line 6). Therefore, we have:

$\left|\mathrm{Pr}[b_{j}\text{ mod }p=0]|=|\mathrm{Pr}[\text{a uniform element in }M\text{ mod }p=0]|\leq (1/p\right)$

Now, for all *j*, we have: $\left|\mathrm{Pr}[\exists j{|}_{1}^{n}\;b_{j}\text{ mod }p=0]|\leq (n/p\right)$, this is negligible in *λ* ($p\geq 2^{2\lambda }$ is large). This means except with probability equal to n/p, which is negligible in *λ*, all of the $b_{j}$ are not 0 and uniform $z_{ij}$ maps to uniform $\left(b_{j}\cdot z_{ij}\right)$ and the reduction works as in the given Algorithm 5.

**Appendix** 4 **: Threat model figure**

Below figure (Fig. 5) indicates that the data server behaves in a semi-honest (honest-but-curious) manner and acts as an adversary in our model.

**Appendix** 5 **: Data representation example**

Table 7 illustrates a sample dataset containing information on three data owners, including their SNP, phenotype, gender, and ethnicity details.

## Appendix B Supplementary material

MMC Proof of Theorem 1. MMC

## Footnotes

1 Physical deletion: the removal of data from the server. Logical deletion: data is retained on the server, but once logically deleted, it will not be retrieved in search queries.
