# Supplementary material for: ACE: A Consent-Embedded privacy-preserving search on genomic database
Source: Heliyon. 2024 Apr 16;10(8):e29399. doi: 10.1016/j.heliyon.2024.e29399 (PMC11636870; doi:10.1016/j.heliyon.2024.e29399)
Supplement: MMC — Proof of Theorem 1. [file mmc1.pdf]

Supplementary material

1 Proof of Theorem 1

Proof. To ensure the security of our scheme, we develop a simulator that accepts as inputs leakage functions $\mathcal{L}^{Stp}(\lambda)$, $\mathcal{L}^{Updt}(add, \{id1, W1\}, \{id2, W2\}, \ldots)$ and query-info, $\mathcal{L}^{Updt}(del, id)$, $\mathcal{L}^{Srch}(w)$ to emulate the Setup, Update, and Search protocols. Our aim is to illustrate that the simulated scheme cannot be distinguished from the real scheme under the non-adaptive attacks. query-info is given to the simulator at the Update phase that gives the information of Delw(id) -for the IDs that are selected by adversary to be deleted- to the simulator at the beginning. Algorithm 8 describes the simulator.
To create the simulator, we will derive multiple games from the real-world game.
Game G_0 G_0 corresponds exactly to the security game depicted in Algorithm 6.

$$\mathbb{P}[\mathrm{Real}_{\mathcal{A}}^{\Sigma}(\lambda) = 1]{=}\mathbb{P}[\mathrm{G}_0 = 1]$$

Game G_1 Instead of using a PRF when generating tags for w and id, G1 selects a new random tag whenever a new combination of w and id is encountered. This tag is then stored in a table for future use. It also does the same for generating $\mathsf{K_w}$ and indices r_{ID}. If an adversary can tell the difference between G0 and G1, this implies that there exists a method to differentiate between PRF F and a genuinely random function. More precisely, there exists an efficient adversary B1 such that

$$\mathbb{P}\left[G_0 = 1\right] - \mathbb{P}\left[G_1 = 1\right] \leq \mathrm{Adv}_{F,B_1}^{\mathrm{prf}}(\lambda)$$

Game G_2 This scenario resembles G1, with the exception that when encrypting the IDs, a constant 0 is encrypted using the symmetric encryption SE. If an adversary A can discern G2 from G1, it implies we could devise an adversary B2 to compromise the IND-CPA security of the standard symmetric key encryption SE.

$$\mathbb{P}\left[G_1 = 1\right] - \mathbb{P}\left[G_2 = 1\right] \leq \mathrm{Adv}_{SE,B_2}^{\mathrm{IND-CPA}}(\lambda)$$

Algorithm 6 Game G_0

Setup This is same as Setup in Algorithm 1

Update-add (a set of IDs with their keywords, $\{\mathsf{ID}_i, \mathbf{W}_{\mathsf{ID}_i}\}$)

1: Parse the set as $(\mathsf{ID}_i, \mathsf{w}_j)$
2: **for** each w **do**
3: $\mathsf{tag_w} \leftarrow F(\mathsf{K}_T, \mathsf{w})$; $\mathsf{K_w} \leftarrow F(\mathsf{K}_S, \mathsf{w})$.//specific ID_i
4: $(\mathsf{ST}_c, c) \leftarrow \mathbf{W}[\mathsf{w}]$
5: **if** $(\mathsf{ST}_c, c) = \perp$ **then**
6: $\mathsf{ST}_0 \xleftarrow{\$} \mathcal{M}, c \leftarrow 0$
7: **end if**
8: **for** $\mathsf{ID}_i \in \mathsf{GDB(w)}$ **do**
9: **if** there is no index r_{ID} in FSet for ID_i **then**
10: Compute index $r_{\mathsf{ID}_i} \leftarrow F(\mathsf{K}_1, \mathsf{ID}_i)$ and a tag $\mathsf{tag}_{\mathsf{ID}_i} \leftarrow F(\mathsf{K}_2, \mathsf{ID}_i)$
11: **end if**
12: Compute $\mathsf{ID}' \leftarrow E(\mathsf{K_w}, \mathsf{ID})$
13: $c \leftarrow c + 1$
14: $\mathsf{ST}_c \leftarrow \pi^{-1}_{\mathsf{SK}}(\mathsf{ST}_{c-1})$; $\mathsf{ST}'_c \leftarrow (\mathsf{ST}_c \bmod p)$
15: $\ell \leftarrow H(k_h, \mathsf{g}^{\mathsf{ST}'_c \cdot \mathsf{tag_w}})$
16: Append ID' to $\mathsf{ISet}[\ell]$
17: Compute $\Delta \leftarrow \mathsf{g}^{\mathsf{ST}'_c \cdot \mathsf{tag_w} / \mathsf{tag}_{\mathsf{ID}_i}}$
18: Append Δ into $\mathsf{FSet}[r_{\mathsf{ID}_i}]$
19: **end for**
20: $\mathbf{W}[\mathsf{w}] \leftarrow (\mathsf{ST}_c, c)$
21: **end for**

Update-del (all entries for a particular ID_i)

1: Compute $\mathsf{tag}_{\mathsf{ID}_i} \leftarrow F(\mathsf{K}_2, \mathsf{ID}_i)$, $r_{\mathsf{ID}_i} \leftarrow F(\mathsf{K}_1, \mathsf{ID}_i)$
2: **for** all elements Δ_i in $\mathsf{FSet}[\mathsf{r}_{\mathsf{ID_i}}]$ **do**
3: Compute $\ell \leftarrow H(k_h, {\Delta_i}^{\mathsf{tag}_{\mathsf{ID}_i}})$
4: Remove corresponding entry from $\mathsf{ISet}[\ell]$ and ℓ
5: **end for**
6: Remove entries of $\mathsf{FSet}[\mathsf{r}_{\mathsf{ID_i}}]$ and $\mathsf{r}_{\mathsf{ID_i}}$

Search

1: Vetter computes $\mathsf{tag_w} \leftarrow F(\mathsf{K}_T, \mathsf{w})$, $\mathsf{tk} \leftarrow \mathsf{g}^{\mathsf{tag_w}}$ and gets $(\mathsf{ST}_c, c) \leftarrow \mathbf{W}[\mathsf{w}]$
2: $\mathsf{RSet} \leftarrow \{\}$
3: **if** $(\mathsf{ST}_c, c) = \perp$ **then**
4: return $\emptyset$
5: **end if**
6: Send $(\mathsf{tk}, \mathsf{ST}_c, c)$ to the server.
Server:
7: **for** $i = c$ to 1 **do**
8: $\ell \leftarrow H(k_h, \mathsf{tk}^{(\mathsf{ST}_i \bmod p)})$
9: $\mathsf{ID}' \leftarrow \mathsf{ISet}[\ell]$
10: $\mathsf{RSet} \leftarrow \mathsf{RSet} \cup \mathsf{ID}'$
11: $\mathsf{ST}_{i-1} \leftarrow \pi_{\mathrm{PK}}(\mathsf{ST}_i)$
12: **end for**
13: **return** RSet

Game G_3 In G_3, the Update phase differs from previous iterations in that it generates the ℓ value using random strings instead of invoking H. During the Search protocol, adjustments are made to the random oracle H such that H(K1, $\mathsf{tk}^{(\mathsf{STc} \bmod p)}$) yields ℓ. Algorithm 7 formally describes G_3 and introduces an intermediate game highlighted in blue. In the pseudocode, we explicitly mark the calls to the random oracle H and monitor the transcripts using the H table.

The purpose of $\hat{G_3}$ is to uphold the coherence of H's transcript: in $\hat{G_3}$, H is never instructed to yield two distinct values for the same input, as indicated by Search-line 8. Instead of promptly generating the ℓ derived from the c-th ST for keyword w from H, $\hat{G3}$ randomly selects them if (STc) has not already appeared in H's transcript. Alternatively, if this situation already exists, $\hat{G3}$ sets ℓ to the preselected value H $\left[K1, \mathsf{g}^{\mathsf{ST'c} \cdot \mathsf{tag_w}}\right]$. Then, $\hat{G_3}$ sets up the random oracle as needed by the Search protocol (line 8) or by a query issued by an adversary (line 5 of H), ensuring that its outputs align with the chosen values of the ℓ's.

By using query-info and getting the information for IDs that are going to be deleted with their keywords that will be searched before deletion (getting the information of Delw in advance), the entries are generated honestly as they are going to be revealed later, and for the not-deleted, not-searched entries, the entries look independent random (line 24). If the adversary is able to distinguish these two games, we can use it to distinguish problem D-ACE. We can use

Algorithm 7 Game G_3, $\hat{G_3}$

Update-add (a set of IDs with their keywords, $\{\mathsf{ID}_i, \mathbf{W}_{\mathsf{ID}_i}\}$)

Parse the set as $(\mathsf{ID}_i, \mathsf{w}_j)$
for each w **do**
  $\mathsf{tag_w} \xleftarrow{\$} \{0,1\}^\lambda$; $\mathsf{K_w} \xleftarrow{\$} \{0,1\}^\lambda$.
  $(\mathsf{ST}_c, c) \leftarrow \mathbf{W}[\mathsf{w}]$
  if $(\mathsf{ST}_c, c) = \perp$ **then**
    $\mathsf{ST}_0 \xleftarrow{\$} \mathcal{M}, c \leftarrow 0$
  end if
  for $\mathsf{ID}_i \in \mathsf{GDB(w)}$ **do**
    if there is no index r_{ID} in FSet for ID_i **then**
      Compute index $r_{\mathsf{ID}_i} \xleftarrow{\$} \{0,1\}^\lambda$ and a tag $\mathsf{tag}_{\mathsf{ID}_i} \xleftarrow{\$} \{0,1\}^\lambda$
    end if
    $\mathsf{ID}' \leftarrow E(\mathsf{K_w}, \{0\}^\lambda)$
    $c \leftarrow c+1$
    $\ell_{ij} \xleftarrow{\$} \{0,1\}^\lambda$
    $\mathsf{ST}_c \leftarrow \pi_{\mathsf{SK}}^{-1}(\mathsf{ST}_{c-1})$; $\mathsf{ST}'_c \leftarrow (\mathsf{ST}_c \bmod p)$
    if $\mathrm{H}(\mathrm{k1}, \mathrm{g}^{\mathsf{ST}'_c \cdot \mathsf{tag_w}}) \neq \perp$ **then**
      bad←true; $\ell_{ij} \leftarrow \mathrm{H}(\mathrm{k1}, \mathrm{g}^{\mathsf{ST}'_c \cdot \mathsf{tag_w}})$
    end if
    if ID_i is in query-info to be deleted and w related to the $\Delta_j \in \{\mathrm{Srch} < \mathrm{Del}\}_i$ **then**
      $\Delta_j \leftarrow \mathrm{g}^{(\mathsf{ST}'_c \cdot \mathsf{tag_w})/\mathsf{tag}_{\mathsf{ID_i}}}$
      program H s.t. $\mathrm{H}(\mathrm{k1}, \mathrm{g}^{\mathsf{ST}'_c \cdot \mathsf{tag_w}}) \leftarrow \ell_{ij}$
      Keep the record of the STs used for w
      else
      $\Delta_j \xleftarrow{\$} \mathbb{G}$
    end if
    Append ID' to $\mathsf{ISet}[\ell]$
    Append Δ into $\mathsf{FSet}[r_{\mathsf{ID}_i}]$
  end for
  $\mathbf{W}[\mathsf{w}] \leftarrow (\mathsf{ST}_c, c)$
end for

H(k,st)

v←H(k,st)
if v=⊥ **then**
  $\mathrm{v} \xleftarrow{\$} \{0,1\}^\lambda$
  if $\exists \mathsf{w}, c$ such that st=$\mathsf{ST}_c \in \mathbf{W}[\mathsf{w}]$ **then**
    bad←true; v← ℓ_{ij}
  end if
  H(k,st)←v
end if
Return v

Update-del (all entries for a particular ID_i)

Use $\mathsf{tag}_{\mathsf{ID}_i}$, r_{ID_i}
for all elements Δ_i in $\mathsf{FSet}[\mathsf{r_{ID_i}}]$ in order **do**
  Compute $\ell \leftarrow H(k1, {\Delta_i}^{\mathsf{tag}_{\mathsf{ID}_i}})$
  Remove corresponding entry from $\mathsf{ISet}[\ell]$ and ℓ
end for
Remove entries of $\mathsf{FSet}[\mathsf{r_{ID_i}}]$ and $\mathsf{r_{ID_i}}$

Search

Use $\mathsf{tag_w}$, $\mathsf{tk} \leftarrow \mathsf{g}^{\mathsf{tag_w}}$ and gets $(\mathsf{ST}_c, c) \leftarrow \mathbf{W}[\mathsf{w}]$
$\mathsf{RSet} \leftarrow \{\}$
if $(\mathsf{ST}_c, c) = \perp$ **then**
  return $\emptyset$
end if
Send $(\mathsf{tk}, \mathsf{ST}_c, c)$ to the server.
Server:
for $i = c$ to 1 **do**
  $\ell \leftarrow H(k1, \mathsf{tk}^{(\mathsf{ST}_c \bmod p)})$
  $\mathsf{ID}' \leftarrow \mathsf{ISet}[\ell]$
  $\mathsf{RSet} \leftarrow \mathsf{RSet} \cup \mathsf{ID}'$
  $\mathsf{ST}_{i-1} \leftarrow \pi_{\mathrm{PK}}(\mathsf{ST}_i)$
end for
return RSet

Algorithm 4 to simulate all the entries to the adversary. The a_i, b_j, c_j in D-ACE correspond to $\mathsf{tag_w}, \mathsf{ST'_c}, \mathsf{tag_{ID}}$ in the G_3, respectively.

$$\mathbb{P}[G_2 = 1] - \mathbb{P}\left[\hat{G_3} = 1\right] \leq \mathrm{Adv}_{B_4}^{\mathrm{D-ACE}}(\lambda)$$

In order to limit the distinguishing advantage between $\hat{G_3}$ and G_3, it becomes evident that if bad is assigned the value true, we can compromise the one-way property of the trapdoor permutation (TDP). To elaborate, we can formulate a reduction B_3 from a distinguisher A inserting N pairs of keywords/documents into the database (refer to [24] for more information).

$$\mathbb{P}\left[\hat{G_3} = 1\right] - \mathbb{P}[G_3 = 1] \leq N \cdot \mathrm{Adv}_{\pi, B_3}^{\mathrm{OW}}(\lambda)$$

Therefore,

Algorithm 8 Simulator

Setup $\left(\mathcal{L}^{Stp}(\lambda)\right)$

```
Initialise empty maps FSet, ISet, W
Select (SK, PK) for π using security parameter λ, and 𝔾 a group of prime order p and generator g.
Send FSet, ISet as EGDB1, EGDB2 to the server.
```

Update-add $\left(\mathcal{L}^{Updt}(add, (id1, id2, \ldots)),\ \text{query-info}\right)$

```
Extract the timestamp of adding the ids from AddHist(set of id) and choose u ← AddHist(set of id)
for i=1 to N_ID do
    Randomly pick index r_IDi ←$ {0,1}^λ and tag_IDi ←$ {0,1}^λ
    for j=1 to NW_IDi do
        ℓ_ij ←$ {0,1}^λ
        if ID_i is in query-info to be deleted and w related to the Δ_j ∈ {Srch < Del}_i then
            ℓ_ij ←$ {0,1}^λ and keep it for this w
            (ST_c, c) ← W[w]
            if (ST_c, c) =⊥ then
                ST_0 ←$ 𝓜, c ← 0
            end if
            c ← c + 1
            ST_c ← π_SK^{-1}(ST_{c-1}); ST'_c ← (ST_c mod p)
            Δ_j ← g^{(ST'_c·tag_w)/tag_IDi} // meaning: Δ_j ← (generated token)^{1/tag_IDi}
            program H s.t. H(k1, g^{ST'_c·tag_w}) ← ℓ_ij
            W[w] ← (ST_0)
            else
            Δ_j ←$ 𝔾
        end if
        Append Δ_j to FSet[r_IDi]
        ID' ← E(K_w, {0}^λ) // (K_w generated randomly for each w and kept in a set for later use)
        Append ID'_ij to ISet[ℓ_ij]
    end for
end for
```

Update-del $\left(\mathcal{L}^{Updt}(del, id)\right)$

```
Extract the timestamps of adding/deleting the id from DelHist(id) and choose u ← u^del in DelHist(id)
Extract the random chosen tag_IDi, and use r_IDi for deleting ID_i
for all elements Δ_j in FSet[r_IDi], use the extracted correlations (Δ_j 2ℓ_ij in Delindex(id)) do
    program H s.t. H(k1, Δ_ij^{tag_IDi}) ← ℓ_ij
end for
Send tag_IDi, and r_IDi as deletion tokens to server
```

Search $\left(\mathcal{L}^{\mathrm{Srch}}(w)\right)$

```
w̄ ← min(sp(w))
Randomly select tag_w or use it if w was in the S_i with {t_Srch < t_Del} in Update
ST_0 ←$ 𝓜 for the ones not in S_i, and c ← 1; (ST_c, c) ← W[w] for w ∈ S_i
for all added IDs (m number of them) in rp(w) at time u in comparison with rp(w) at time u-1 do
    for i=c to c+m-1 do
        skip the skipped tokens from the leakage (indices got deleted before being searched) by computing ST_i ← π_SK^{-1}(ST_{i-1})
        Compute ST_i ← π_SK^{-1}(ST_{i-1}) for non-deleted ones
        ST'_i ← (ST_i mod p)
        program H s.t. H(k1, g^{ST'_i·tag_w}) ← ℓ_ij //use TimeDB[w] to extract set of ℓs
    end for
end for
ST_m ← W[w̄]
Send (g^{tag_w}, ST_m) to the server.
```

$$\mathbb{P}[G_2 = 1] - \mathbb{P}[G_3 = 1] \leq N \cdot \mathrm{Adv}^{\mathrm{OW}}_{\pi, B_3}(\lambda) + \mathrm{Adv}^{\mathrm{D-ACE}}_{B_4}(\lambda)$$

Game G_4 During the Search phase, G_4 constructs the search token from ST_0 by iterating Π instead of utilizing a previously computed and stored token. Additionally, if an entry is accessed for the first time, the game randomly selects it from $\mathcal{M}$. This happens for all STs except the ones that have been used for the tags related to the query-info IDs.

$$\mathbb{P}[G_3 = 1] - \mathbb{P}[G_4 = 1] = 0$$

The simulator It is detailed in Algorithm 8. Instead of employing the keyword w, the Simulator utilizes $w = \min \mathrm{sp(w)}$ as the counter, which is uniquely assigned from w through the utilization of the leakage function.

$$\mathbb{P}\left[G_4 = 1\right] - \mathbb{P}\left[\mathrm{Ideal}^{\Sigma}_{\mathcal{A},\mathcal{S}}(\lambda) = 1\right] = 0$$

Finally, we can conclude:

$$\mathbb{P}[\mathrm{Real}^{\Sigma}_{\mathcal{A},\mathcal{S}}(\lambda) = 1] - [\mathbb{P}\left[\mathrm{Ideal}^{\Sigma}_{\mathcal{A},\mathcal{S}}(\lambda) = 1\right] \leq$$
$$\mathrm{Adv}^{\mathrm{prf}}_{F,B_1}(\lambda) + \mathrm{Adv}^{\mathrm{IND-CPA}}_{SE,B_2}(\lambda) + N \cdot \mathrm{Adv}^{\mathrm{OW}}_{\pi,B_3}(\lambda) + \mathrm{Adv}^{\mathrm{D-ACE}}_{B_4}(\lambda)$$
